# Inhibition of NF-κB transcriptional activation in HepG2 cells by diterpenoids from the soft coral *Sinularia maxima*

**DOI:** 10.1007/s12272-013-0230-3

**Published:** 2013-08-10

**Authors:** Nguyen Phuong Thao, Nguyen Hoai Nam, Nguyen Xuan Cuong, Bui Thi Thuy Luyen, Bui Huu Tai, Ji Eun Kim, Seok Bean Song, Phan Van Kiem, Chau Van Minh, Young Ho Kim

**Affiliations:** 1 Institute of Marine Biochemistry, Vietnam Academy of Science and Technology (VAST), 18 Hoang Quoc Viet, Nghiado, Caugiay, Hanoi, Vietnam; 2 College of Pharmacy, Chungnam National University, Daejeon, 305-764 Korea

**Keywords:** Soft coral, *Sinularia maxima*, Nuclear factor-κB, HepG2 cell, ICAM-1, iNOS

## Abstract

Anti-inflammatory transcriptional effects of nineteen compounds (**1**–**19**) from the soft coral *Sinularia maxima* were evaluated using NF-κB luciferase and reverse transcriptase polymerase chain reaction. Compounds **1**, **2**, **4**, **8**, **15**, **17**, and **18** significantly inhibited TNFα-induced NF-κB transcriptional activity in HepG2 cells in a dose-dependent manner, with IC_50_ values ranging from 15.81 ± 2.29 to 29.10 ± 1.54 μM. Furthermore, the transcriptional inhibitory function of these compounds was confirmed by a decrease in intercellular adhesion molecule-1 and inducible nitric oxide synthase gene expression levels in HepG2 cells. These results provide a scientific rationale for the use of the soft coral *S. maxima* warrant further studies to develop new agents for the prevention and treatment of inflammatory.

## Introduction

Activation of nuclear factor kappa B (NF-κB) represents a family of Rel domain-containing proteins including five NF-κB units that can form 15 transcription factors through homo- and heterodimerization (Baldwin 2001a). NF-κB plays an important role in the transcriptional regulation of numerous cytokines and adhesion molecules. It is arguably the most important transcription factor for the initiation or progression of numerous human diseases (Mattson and Camandola 2001). As a ubiquitous transcription factor governing the expression of viruses or a variety of inflammatory cytokine genes, NF-κB was first implicated in the pathogenesis of human immunodeficiency virus-1 (HIV-1) infection (Nabel and Baltimore 1987). NF-κB, a nuclear transcription factor, regulates the expression of various genes, including cytokines, iNOS, COX-2 and ICAM-1, which play critical roles in apoptosis, autoimmune diseases, and inflammation (Albert and Baldwin 1996). Further studies suggest that activation of NF-κB is responsible for the pathological progression of neurological disorders, carcinogenesis, immune deficiency, rheumatoid arthritis, atherogenesis, Crohn’s disease, cystic fibrosis, asthma, osteopetrosis, ischemic reperfusion, etc. (Chen et al. 1999).

**Fig. 1:**
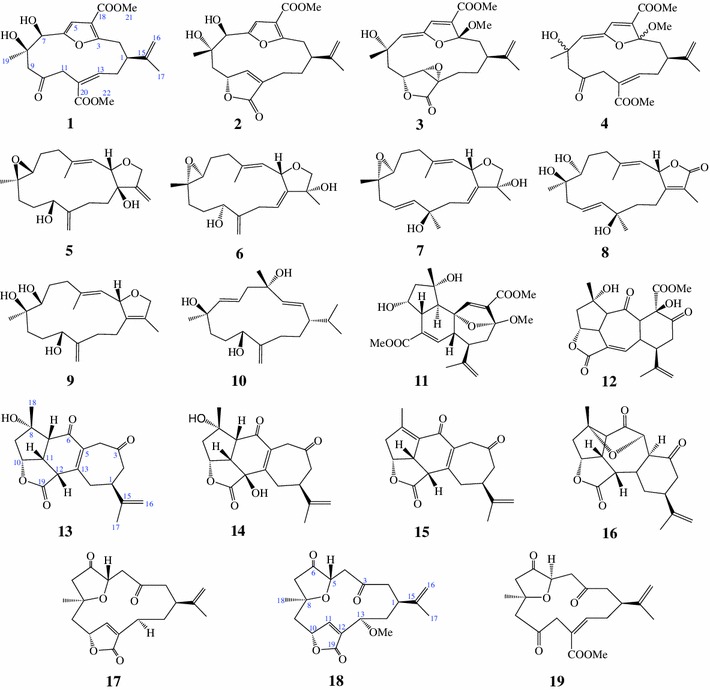
Structure of compounds **1**–**19** from the soft coral *Sinularia maxima*

Among the Alcyonacean soft corals, genus *Sinularia* is one of the most widely distributed soft coral genera, constituting a dominant portion of the biomass in the tropical reef environment (Lakshmi and Kumar 2009). Previous studies have indicated that diterpenes, a main constituent of the genus *Sinularia*, exhibit various biological activities, such as anti-inflammatory (Chao et al. 2011; Cheng et al. 2010; Lu et al. 2010; Su and Wen 2011), antiviral (Cheng et al. 2010), and cytotoxic (Grote et al. 2008; Kamel et al. 2007; Lo et al. 2009; Su et al. 2009) effects. As a part of our ongoing investigations on screening active compounds from Vietnamese *Sinularia* soft corals towards anti-inflammatory effects (Thao et al. 2012, 2013a, b), we recently reported the isolation, structure elucidation, and inhibitory effects on lipopolysaccharide-stimulated production of proinflammatory cytokines in bone marrow-derived dendritic cells of 12 diterpenoids [sinumaximol A (**1**), sinumaximol B (**2**), sinumaximol C (**3**), sethukarailin (**4**), sinumaximol D (**5**), sinumaximol E (**6**), sinumaximol F (**7**), sinumaximol G (**8**), sinumaximol H (**9**), (1*S*,2*E*,4*S*,6*E*,8*S*,11*R*)-2,6,12(20)-cembratriene-4,8,11-triol (**10**), isomandapamate (**11**), and sinumaximol I (**12**)] (Thao et al. 2012) and 7 norditerpenoids [scabrolide A (**13**), 12-hydroxy-scabrolide A (**14**), yonarolide (**15**), ineleganolide (**16**), 5-epinorcembrene (**17**), 13-*epi*-scabrolide C (**18**), and norcembrene 5 (**19**)] (Thao et al. 2013a) from *Sinularia maxima* (see Fig. 1). The current study provides new insight into the ways by which diterpenoids and norditerpenoids modulate TNFα-induced NF-κB activity in human HepG2 cells.

## Materials and methods

### Biological material

The sample of soft coral *S. maxima* was collected at Nhatrang Bay, in November in 2010 and identified by Prof. Do Cong Thung (Institute of Marine Environment and Resources, VAST). A voucher specimen (SM112010_01) was deposited at the Institute of Marine Biochemistry and Institute of Marine Environment and Resources, VAST.

### Cell culture and reagents

Human hepatocarcinoma HepG2 cells were maintained in Dulbecco’s modified Eagle’s medium (Invitrogen, Carlsbad, CA, USA) containing 10 % heat-inactivated fetal bovine serum, 100 units/mL penicillin, and 10 μg/mL streptomycin at 37 °C and 5 % CO_2_. Human TNFα was purchased from ATgen (Seoul, Korea). Cells were counted with a hemocytometer, and the number of viable cells was determined through trypan blue dye exclusion.

### Cytotoxicity assay

A 3-(4,5-dimethylthiazol-2-yl)-5-(3-carboxymethoxyphenyl)-2-(4-sulfophenyl)-2*H*-tetrazolium, inner salt (MTS) assay (CellTiter 96^®^ AQueous One Solution Assay, Promega, Madison, WI, USA) was performed to analyze the effect of the different compounds on cell viability. Cells were cultured overnight in 96-well plates (1 × 10^4^ cells per well). Cell viability was assessed after the incubation with the compounds at a concentration of 10 μM for 24 h. The number of viable cells was determined by measuring the absorbance at 490 nm of the dissolved formazan product after addition of MTS for 30 min as described by the manufacturer.

### NF-κB and iNOS-luciferase assay

The luciferase vector was first transfected into HepG2 cells. After a limited amount of time, the cells were lysed and luciferin, the substrate of luciferase, was introduced into the cellular extract along with Mg^2+^ and an excess of ATP. Under these conditions, luciferase enzymes expressed by the reporter vector could catalyze the oxidative carboxylation of luciferin. Cells were seeded at 1.5 × 10^5^ cells per well in a 12-well plate and grown for 24 h. All cells were transfected using Lipofectamine™ LTX (Invitrogen) according to the manufacturer’s protocol. Luciferase (Luc) activity was assayed using an LB 953 Autolumat (EG&G Berthold, Nashua, NH, USA) as described previously (Kim et al. 2010). NF-κB-Luc was kindly provided by Dr. Kyoon E. Kim (Chungnam National University, Daejeon, Korea). The transfected HepG2 cells were pretreated for 1 h with either vehicle (DMSO) and compounds, followed by 1 h of treatment with 10 ng/mL TNFα. Unstimulated HepG2 cells were used as a negative control (−). Cells were then harvested, and luciferase activity was assayed. All experiments were performed in triplicate.

### RNA preparation and RT-PCR

HepG2 cells were pretreated in the absence and presence of compounds for 1 h, then exposed to 10 ng/mL TNFα for 6 h. Total mRNA was prepared from the cell pellets using Easy-blue. The levels of mRNA were assessed by reverse transcriptase polymerase chain reaction (RT-PCR) (Quang et al. 2012).

Total RNA was extracted from cells using easy-BLUE™ (iNtRON Biotechnology, Seoul). Approximately 2 μg of total RNA was reverse-transcribed using Moloney murine leukemia virus reverse transcriptase and oligo (dT) primers (Promega) for 1 h at 42 °C. The resulting cDNA was polymerase chain reaction-amplified using Taq polymerase premixture (TaKaRa, Shiga, Japan). Polymerase chain reaction products were subjected to electrophoresis on 1 % agarose gels and stained with ethidium bromide. Polymerase chain reaction was conducted with the following primer pairs: iNOS sense 5′-TCATCCGCTATGCTGGCTAC-3′, iNOS antisense 5′-CTCAGGGTCACGGCCATTG-3′, ICAM-1 sense 5′-GCCCAGCACTTCACGCATCAG-3′, ICAM-1 antisense 5′-GACCAGGCACCAGACCAAAGACC-3′, glyceraldehyde 3-phosphate dehydrogenase sense 5′-TGTTGCCATCAATGACCCCTT-3′, and glyceraldehyde 3-phosphate dehydrogenase antisense 5′CTCCACGACGTACTCAGCG-3′. β-actin sense 5′-TCACCCACACTGTGCCCATCTACG-3′, and β-actin antisense 5′-CAGCGGAACCGCTCATTGCCAATG-3′.

### Statistical analysis

All results are expressed as mean±SD values. Data were analyzed by one-factor analysis of variance. Quantification of polymerase chain reaction products was performed using Image Lab™ software (Bio-Rad). If a statistically significant effect was found, the Newman–Keuls test was performed to isolate the difference between the groups. *P* < 0.5 was considered to be significant.

## Results

To investigate cellular toxicity of the compounds **1–19**, they were applied at various concentrations to HepG2 cells for 24 h, after which cell viability was measured in an MTS assay as described in Materials and methods. None of the compounds displayed any cellular toxicity at the concentration of 10 μM (data not shown). They were therefore used in subsequent experiments for further evaluation of their effects on NF-κB activation, iNOS and ICAM-1 expressions at concentrations of 0.1, 1.0 and 10 μM (see Fig. 4).

**Fig. 2:**
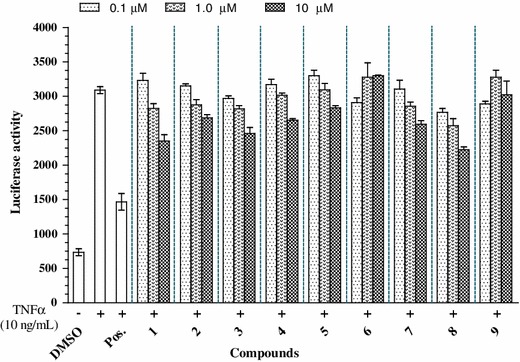
Effects of compounds **1**–**9** on tumor necrosis factor-α (TNFα)-induced nuclear transcription factor κB activation in HepG2 cells. HepG2 cells transiently transfected with pNF-κB-luciferase were pretreated for 1 h with vehicle (dimethyl sulfoxide-DMSO) or one of the compounds, prior to 1 h of treatment with TNFα (10 ng/mL). Unstimulated HepG2 cells acted as a negative control. Cells were then harvested, and luciferase activities were assessed. Results are expressed as relative luciferase activity. Sulfasalazine was used as a positive (Pos.) control. Data are mean–SD values (*n* = 3). *P* < 0.5 versus control

**Fig. 3:**
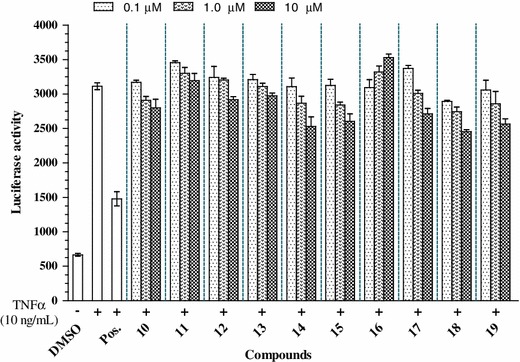
Effects of compounds **10**–1**9** on tumor necrosis factor-α (TNFα)-induced nuclear transcription factor κB activation in HepG2 cells. HepG2 cells transiently transfected with pNF-κB-luciferase were pretreated for 1 h with vehicle (dimethyl sulfoxide-DMSO) or one of the compounds, prior to 1 h of treatment with TNFα (10 ng/mL). Unstimulated HepG2 cells acted as a negative control. Cells were then harvested, and luciferase activities were assessed. Results are expressed as relative luciferase activity. Sulfasalazine was used as a positive (Pos.) control. Data are mean–SD values (*n* = 3). *P* < 0.5 versus control

To evaluate the anti-inflammatory activity of nineteen compounds listed above, we first examined their inhibitory effects on NF-κB transcriptional activation in HepG2 cells (see Figs. 2, 3). Cells were treated with compounds at various concentrations prior to stimulation with TNFα (10 ng/mL). Among compounds tested, **1**, **2**, **4**, **8**, **15**, **17**, and **18** were found to have effect on the inhibition of NF-κB activation with 50 % inhibition concentration (IC_50_) values ranging from 15.81 ± 2.29 to 29.10 ± 1.54 μM. Other compounds exhibited moderate, weak or not determined activities (Table 1).

**Table 1:** Effects of compounds **1**–**19** on NF-κB luciferase activity in HepG2 cells

Compound	IC_50_ values (μM)
**1**	21.35 ± 3.21
**2**	29.10 ± 1.54
**3**	40.49 ± 2.07
**4**	25.81 ± 1.38
**5**	50.42 ± 2.11
**6**	ND
**7**	60.33 ± 0.88
**8**	15.81 ± 2.29
**9**	ND
**10**	224.05 ± 5.89
**11**	325.71 ± 4.77
**12**	40.42 ± 2.11
**13**	75.22 ± 3.66
**14**	45.12 ± 1.75
**15**	25.1 ± 2.58
**16**	ND
**17**	28.19 ± 2.65
**18**	20.13 ± 0.29
**19**	84.80 ± 4.34
Positive control^a^	0.90 ± 0.20

*IC*_*5*0_ 50 % inhibition concentration, *ND* not determined

^a^Sulfasalazine was used as positive control compound

## Discussion

NF-κB was first described in 1986 as a nuclear transcription factor required for immunoglobulin kappa light chain transcription in B-cells. Since its discovery, it has been demonstrated that NF-κB is constitutively expressed in all cell types and plays a central role as a regulator of the cellular stress response. The NF-κB-mediated signaling pathway has been considered both pro-inflammatory and anti-apoptotic in character, and therefore, has been implicated in the pathogenesis of a wide variety of diseases, including inflammatory disorders and tumor development (Robinson and Mann 2010). As previously demonstrated, activation of NF-κB has been linked to multiple pathophysiological conditions such as cancer, arthritis, asthma, inflammatory bowel disease, and other inflammatory conditions (Baldwin 2001b). It can be activated by various stimuli, such as microbial and viral products, cytokines, DNA damage, and noxious chemicals. The induction of several pro-inflammatory mediators occurs as a result of increased inducible nitric oxide synthase (iNOS) and cyclooxygenase-2 (COX-2) activities (Surha et al. 2001). NF-κB and the signaling pathways that regulate many physiological processes, including innate and adaptive immune responses, cell death, and inflammation, have become a focal point for intense drug discovery and development efforts (Chung et al. 2007; Perkins 2007). Indeed, increasing evidence has validated NF-κB as a target for anti-inflammatory and anticancer agents.

To date, the inhibition of components from *S. maxima* on NF-κB transcriptional activation has not been evaluated. In this study, the effects of compounds **1**–**19** on TNFα-induced NF-κB transcriptional activity in HepG2 cells were evaluated using a NF-κB luciferase assay. To confirm their inhibitory effects of the compounds on NF-κB transcriptional activity, the effects of the isolated compounds on the upregulation of the pro-inflammatory proteins iNOS and ICAM-1 were assessed in TNFα-stimulated HepG2 cells by RT-PCR.

HepG2 cells were first transfected with NF-κB luciferase reporter plasmids. After treatment with 10 ng/mL TNF-α, luciferase activity increased fivefold, demonstrating an increase in transcriptional activity compared to untreated cells. Compounds were pretreated with transfected HepG2 cells at various concentrations, followed by stimulation with TNFα. The results showed that compounds **1**, **2**, **4**, **8**, **15**, **17**, and **18** significantly inhibited TNFα-induced NF-κB transcriptional activation in a dose-dependent manner with IC_50_ values of 21.35 ± 3.21, 29.10 ± 1.54, 25.81 ± 1.38, 15.81 ± 2.29, 25.1 ± 2.58, 28.19 ± 2.65, 20.13 ± 0.29 μM, respectively (see Figs. 2, 3). Compounds **3**, **10**–**14**, and **19** exhibited moderate or weak inhibitory effects with IC_50_ values ranging from 40.42 ± 2.11 to 325.71 ± 4.77 μM, whereas other compounds were inactive at the indicated concentrations, compared with the positive control (see Table 1).

NF-κB activation is known to be involved in the upregulation of inflammatory NF-κB target gene expression, including iNOS and ICAM-1, which play important roles in the inflammatory response. iNOS is highly expressed in macrophages, leading to organ destruction in some inflammatory and autoimmune diseases (Kleinert et al. 2004). ICAM-1 (CD54) is a 90-kDa inducible cell-surface glycoprotein that promotes leukocyte adhesion in inflammatory conditions (Rothlein et al. 1986; Springer 1990). Analysis of the ICAM-1 complementary deoxyribonucleic acid sequence has revealed it to be a member of the immunoglobulin gene superfamily (Staunton et al. 1988). ICAM-1 is expressed basally at low levels on many cell types, including endothelial cells, macrophages, myocytes, and vascular smooth muscle cells, but can be induced to high levels by stimulation with LPS, phorbol ester, or inflammatory cytokines, such as TNF-α or interleukin-1β (Colic and Drabek 1991; Ruetten et al. 1999).

**Fig. 4:**
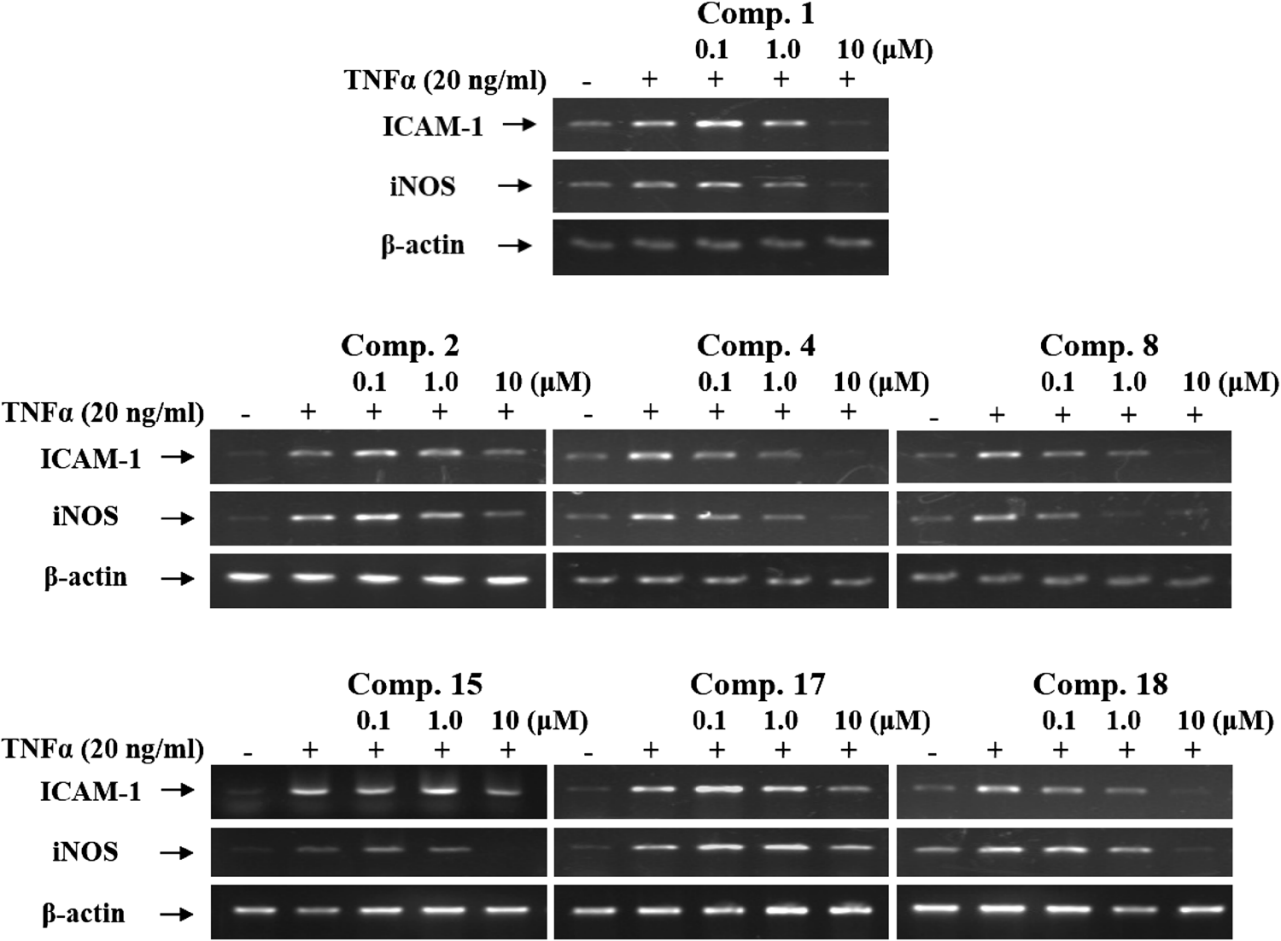
Inhibitory effects of compounds **1**–**19** on TNFα-induced expression of inducible nitric oxide synthase (iNOS) and intercellular adhesion molecule-1 (ICAM-1) mRNAs in HepG2 cells. Effects of compounds **1**, **2**, **4**, **8**, **15**, **17** and **18** on ICAM-1 and iNOS mRNA expression in HepG2 cells were assessed. HepG2 cells were pretreated with one of the listed compounds for 1 h and then treated with TNFα (10 ng/mL) for 6 h. Total mRNAs were prepared from the cell pellets using easy-BLUE™ (iNtRON Biotechnology, Seoul)

To confirm the transcriptional inhibitory function of compounds **1**, **2**, **4**, **8**, **15**, **17**, and **18**; we further investigated their effects on ICAM-1 and iNOS gene expression in TNFα-stimulated HepG2 cells using RT-PCR. Consistent with their inhibitory activity toward NF-κB, compounds **1**, **2**, **4**, **8**, **15**, **17**, and **18** significantly inhibited the induction of ICAM-1 and iNOS mRNA in a dose-dependent manner (see Fig. 4), indicating that these compounds reduced transcription of these genes. Moreover, the housekeeping protein β-actin was unchanged by the presence of compounds **1**, **2**, **4**, **8**, **15**, **17**, and **18** at the same concentration (see Fig. 4). Our data suggest that compounds **1**, **2**, **4**, **8**, **15**, **17**, and **18** isolated from the soft coral *S. maxima* have therapeutic potential as anti-inflammatory, anti-atherosclerotic, and anti-arthritic substances. However, elucidation of the detailed mechanisms underlying the inhibition of the TNFα-induced NF-κB pathway and subsequent decreases in ICAM-1 and iNOS gene expression by compounds **1**, **2**, **4**, **8**, **15**, **17**, and **18** requires further investigation.

Consideration of the structure–activity relationship of these compounds indicated that the ketone groups at C-3 and/or C-6, the hydroxyl group at C-7 and/or C-8 are necessary for the anti-inflammatory activity of diterpenoids and norditerpenoids. This finding was confirmed by comparing the structure and activity of compounds **1**, **2**, **4**, **8**, **15**, **17**, and **18**, therefore this information may facilitate identification of anti-inflammatory lead compounds from diterpenoids and norditerpenoids. This primary finding provides support for further studies on these compounds for the development of anti-inflammatory agents.
